# Passive social media use and depression among college students during public health emergencies: a chain mediation approach

**DOI:** 10.3389/fpsyg.2025.1627637

**Published:** 2025-07-22

**Authors:** Lin Wang, Shuhe Li, Yajing Liu, Lu Cheng

**Affiliations:** ^1^Chinese Academy of Sciences and Education Evaluation, Hangzhou Dianzi University, Hangzhou, China; ^2^Jiangxi Institute of Fashion Technology, Nanchang, China; ^3^Beijing Municipal Commission of Transport, Beijing, China

**Keywords:** public health emergencies, passive social media usage, vicarious traumatization, fear of missing out, depression, COVID-19 pandemic

## Abstract

**Introduction:**

Despite growing evidence linking passive social media use to depression during public health emergencies, the underlying psychological mechanisms remain unclear. This study investigates the impact of passive social media use on depression among college students during the COVID-19 pandemic, specifically examining the mediating roles of vicarious traumatization and fear of missing out (FoMO).

**Methods:**

A structured survey was conducted among 322 college students. Data were collected through questionnaires and analyzed using SPSS 23.0. ANOVA, linear regression, and Bootstrap methods were employed to assess relationships and mediating effects in a model linking passive social media use, vicarious traumatization, FoMO, and depression.

**Results:**

Results indicated that passive social media use did not directly predict depression, suggesting possible masking effects. However, passive social media use significantly predicted depression through partial mediation by vicarious traumatization and FoMO. Additionally, a chain mediation pathway was identified, in which vicarious traumatization and FoMO sequentially mediated the relationship between passive social media use and depression.

**Discussion:**

These findings reveal how passive social media behaviors contribute to depression during public health crises by highlighting the cognitive-psychological processes involved. The study advances understanding of social media’s negative effects and provides actionable recommendations for mental health interventions during emergencies.

## Introduction

Social media has become a pervasive element in daily life, yet its impact on mental health, particularly depression, remains complex and multifaceted. Research has shown mixed results: while active social media use has been linked to reduced depressive symptoms in younger individuals, passive social media use may increase such symptoms (Lewin et al., 2023). While for older adults, study revealed that active social media use is associated with higher odds of depressive symptoms, passive social media use is linked to lower odds (Lewin et al., 2023). Valkenburg et al. (2022) found limited support for the hypotheses that active social media use leads to positive well-being and passive social media use leads to negative well-being, suggesting that the characteristics of social media use, such as content valence and user motivations, should be considered. Longitudinal research by Primack et al. (2021) indicates that initial social media use can predict subsequent depression, supporting a temporal association that suggests causality. In contrast, Hartanto et al. (2021) emphasize reverse causation, where depressive symptoms may drive social media use as a compensatory mechanism to fulfill psychosocial needs. Vidal et al. (2020) identified themes such as the quantity and quality of social media use, social aspects, and mental health symptom disclosure, finding that problematic social media use is associated with emotional maladjustment and depressive mood. Collectively, these studies underscore the intricate relationship between social media use and depression, indicating that the context, content, and motives of social media use play crucial roles in determining its impact on mental health. This is especially relevant during public health emergencies.

Public health emergencies frequently lead to profound shifts in the public’s information-seeking behavior and have a substantial impact on mental health (Bento et al., 2020). During public health emergencies, the way social media is utilized has a profound impact on individual mental health (Drouin et al., 2020). Research indicates that social media not only serves as a crucial channel for accessing information but also plays a pivotal role in shaping users’ emotions and behaviors (Ngien and Jiang, 2022). College students, who are already navigating a challenging period of life transitions, have found themselves increasingly reliant on social media for updates, connection, and emotional support during these times of uncertainty (Cauberghe et al., 2021). While social media provides a valuable means for staying informed and connected, research indicates that passive social media use, where individuals consume content without actively engaging, can negatively impact mental well-being. Specifically, this type of usage often results in information overload and emotional exhaustion, which can exacerbate mental health issues such as depression (Lee et al., 2023). Public health crises, such as the COVID-19 pandemic, have profoundly altered how individuals interact with digital platforms, particularly social media (Wu et al., 2025). During these times, university students, isolated by quarantine and social distancing measures, increasingly turned to social media to stay connected and informed. However, excessive passive consumption, browsing content without active engagement, often exposed them to overwhelming negative information, adversely affecting their emotional well-being and mental health (van der Schuur et al., 2019). The COVID-19 pandemic serves as a clear example of this dynamic. During the COVID-19 pandemic, social media emerged as a critical platform for information dissemination. A survey by Fan et al. (2020) revealed that social media was the primary source of pandemic-related information, with 50.7% of the Chinese public using platforms like QQ and WeChat to stay informed. Yet, the lack of interaction during such passive browsing behaviors has been linked to negative emotions, such as anxiety and stress (Qian et al., 2020).

College students, as active digital users, exhibited significantly higher social media usage during public health emergencies, making them particularly susceptible to passive usage behaviors (Yue et al., 2022). Passive scrolling through posts, images, and news can intensify exposure to distressing information, amplifying negative emotions like anxiety and stress and potentially contributing to elevated levels of depression. This underscores the dual-edged nature of social media use during crises, where the benefits of staying informed may come at a cost to mental health.

Zhu et al. (2023) examined the relationship between depression and social media use in the information system field through systematic review. Despite the growing prevalence of passive social media use behavior, previous studies have primarily employed cross-sectional surveys and similar methods to explore individuals’ passive social media use and its influencing factors (Zhu et al., 2023), the specific mechanisms by which this behavior influences depression in college students during public health emergencies remain underexplored. Previous study has not fully explored to what extent and through what factors passive social media information behavior affects depression in such a situation. Therefore, it is essential to clarify the impact mechanism and how it evolves during such emergencies. Understanding the psychological impact of this behavior is crucial to develop interventions that promote healthier social media habits and improve mental health outcomes. We draw on previous research highlighting the mental health implications of social media use during the pandemic and the prevalence of depression among Chinese college students to contextualize our investigation (Luo et al., 2021). This study aims to investigate the effect of passive social media usage on the depression levels of college students in the context of the COVID-19 pandemic. It mainly addresses the following two research questions (RQ):

*RQ1*: How does passive social media usage impact depression among college students?

*RQ2*: What mediating factors influence the relationship between passive social media usage and depression levels in college students?

By answering these questions, this research seeks to shed light on the underlying processes that link passive social media consumption to depression, providing valuable insights for mental health professionals and educators. The novelty of this study lies in conceptualizing and operationalizing the dark side of social media information behavior, specifically in the context of public health emergencies, with a focus on its impact on psychological well-being. By integrating passive social media information behavior with concepts like vicarious traumatization that emerge during public crises, this study offers a novel perspective on the mechanisms underlying depression development in the mobile media context. This study aims to provide a deeper insight into the mediating effects of vicarious traumatization and FoMO as intermediate variables that lie in the pathway from passive social media usage to the depression level. This study contributes to the existing literature by shedding light on the cognitive, psychological, and social processes underlying the dark side of online information behavior and cyberpsychology. These areas have gained increasing significance in research, particularly in response to the COVID-19 pandemic and the associated “infodemic.”

## Literature review and research hypotheses

The impact of social media on mental health has become a critical research focus in the field of public health. Existing literature explores the effects of various social media usage patterns on the mental health, emotional states, and behaviors of different populations from multiple perspectives. Lee and Cho (2019) highlighted that social media usage, through its provision of instrumental and informational support, is negatively associated with poor mental health outcomes. This finding not only underscores the positive role of social media in supporting the mental health of vulnerable groups but also raises concerns about the potential adverse effects of passive social media use, such as information overload. Wang and Liu (2016) further revealed how public opinion leaders and media outlets can reinforce stereotypes about depression, thereby influencing users’ attitudes and mental health. This provides theoretical support for examining the impact of negative information on the mental health of university students. Ngien and Jiang (2022) identified psychological mechanisms, such as fatalism and information fatigue, through which social media indirectly affects stress levels in young people, aligning closely with our hypothesis that passive social media use may lead to information overload and emotional exhaustion. Chen et al. (2023) investigated how passive social media use, while potentially mitigating pandemic fatigue through information overload, may simultaneously trigger negative psychological effects. This offers valuable insights for exploring the relationship between passive social media use and depression. Additionally, Cho et al. (2024) emphasized the complex emotional regulation pathways facilitated by social media during crises, suggesting the need to focus on its role in emotional regulation during public health emergencies. Together, these studies provide critical theoretical foundations and insights for investigating how passive social media use during public health emergencies impacts the mental health of college students.

### The relationship between passive social media use and depression

As mobile social media usage has become increasingly widespread, research has focused on the relationship between social media use and its impact on users’ mental health (Primack et al., 2017). The use of mobile social media can be classified into active usage behavior and passive usage behavior. The former refers to information-producing activities that promote online communication (e.g., updating status, commenting on posts). The latter refers to browsing behavior characterized by a lack of interaction with others, e.g., browsing news or story highlights. Excessive passive behaviors like browsing can contribute to negative emotions and diminish users’ overall life satisfaction (Yao et al., 2014; Huang, 2017). Research has found that when jealousy and upward social comparison are introduced as sequential mediators, passive social media use has a significant positive impact on depression levels (Li, 2018). The frequency of passive social media usage can also negatively predict self-esteem through the mediating effect of upward social comparison, while self-esteem is negatively correlated with anxiety and depression (Liu et al., 2017a). Another detrimental effect of passive social media use is the heightened risk of self-concept disorders. Individuals with a weak self-concept are more susceptible to external pressures, which can ultimately lead to depression. Yang et al. (2007) pointed out that passive social media usage positively predicts depression levels through the mediating effect of rumination. The chain mediation effect of rumination and core self-evaluation suggests that the frequency of passive social media use positively contributes to higher levels of depression. In addition, passive social media usage reduces individual well-being (Liu et al., 2017b).

During the COVID-19 pandemic, quarantine and isolation measures confined people to their homes, reducing the frequency of their outings. Universities either delayed reopening or shifted to online education, and holiday periods were extended. College students inevitably have more spare time to use mobile phones and browse social media. They are experiencing a more serious “infodemic” (information pandemic). Social media has become the main source for college students to obtain information about the pandemic; however, excessive browsing can heighten anxiety, sadness, and worry, ultimately harming their mental health. Therefore, the following hypotheses could be posited:

*H1*: The frequency of passive social media use among college students has a significant positive effect on their levels of depression.

### The mediating effect of vicarious traumatization

Vicarious traumatization (VT) originates from the constructivist self-development theory. It refers to the traumatic experience of psychotherapists in serving patients (McCann and Pearlman, 1990). The concept of vicarious trauma has been widely used in various contexts, and its conceptual connotation has been enriched. Vicarious traumatization refers to indirect exposure to a severe traumatic event through activities such as watching, listening, reading, or discussing information about the event, which can evoke psychological responses similar to those experienced by individuals who have directly endured the trauma (Byrne et al., 2006). Vicarious trauma has numerous adverse effects on individuals, including a decline in self-efficacy, impaired cognitive processes, and diminished sense of security, trust, and self-esteem (McCann and Pearlman, 1990). Ma reported that individuals experiencing vicarious trauma may exhibit symptoms such as inattention, fatigue, reduced physical performance, and emotional instability. These symptoms are often accompanied by mental health issues, with depression being the most prominent. Silver et al., (2013) observed that prolonged focus on negative news from the media can negatively affect both physical and mental health, potentially leading to vicarious trauma in severe cases.

During public health emergencies like COVID-19 pandemic, social media is flooded with negative information, including reports of illness, mass infections, deaths, broken families, pleas for help from affected areas, and other tragic stories related to the crisis. Users are immersed in such information environments, where the frequency of browsing far exceeds that of content creation. Their passive social media use has become more frequent than before. The availability of negative pandemic information in multimedia formats can make users feel as though they are experiencing the situation first-hand. Coupled with the impact of empathy, this can lead to virtual emotional interactions with those directly affected, ultimately resulting in vicarious trauma (Xu and Yang, 2009). It, in turn, leads to depression. These adverse effects may be more pronounced in college students since they lack social and life experiences. Therefore, we hypothesize that:

*H2*: Vicarious traumatization mediates the relationship between the frequency of passive social media use and depression levels among college students.

### The mediating effect of fear of missing out

Cognitive psychologist Przybylski *et al*. were pioneers in exploring FoMO (Fear of Missing Out) on social media, defining it as the pervasive anxiety that occurs when individuals feel they are missing out on experiences that they desire to be a part of due to their absence (Przybylski et al., 2013). It is primarily characterized by a continuous desire to know what others are doing. Przybylski et al. (2013) also developed a FoMO measurement scale. In essence, FoMO refers to the discomfort or anxiety stemming from missing out on the activities and experiences of friends.

In traditional society, individuals had limited means to stay informed about others’ activities. However, with the development of mobile internet and the widespread use of social media, people can now check what others are doing anytime and anywhere. This constant connectivity has heightened the fear of missing out. Zhao et al. (2017) identified that the intensity, frequency, and persistence of mobile social media use can reflect users’ FoMO levels. Beata (2013) also reported a positive correlation between FoMO and the frequency of checking messages on mobile platforms. Research has shown that passive social media usage can negatively impact individuals’ mental health, including fear of missing out. Ma (2020) maintained that passive social media usage is important in generating FoMO. Wortham (2011) revealed that FoMO is a contributing factor to negative emotions, including depression.

During the COVID-19 pandemic, social media provided regular updates on the progression of the crisis. As highly educated individuals, college students were particularly attentive to the situation, which inadvertently increased their passive social media usage. This makes college students more susceptible to FoMO. Additionally, pandemic information comes from diverse sources with varying levels of accuracy, mixing false or misleading information with factual reports. Such an infodemic exacerbates FoMO symptoms in students and may heighten their levels of depression. Therefore, the following hypothesis could be posited:

*H3*: FoMO mediates the relationship between the frequency of passive social media use and depression levels in college students.

### The chain mediating effect of vicarious traumatization and FoMO

**Figure 1:**
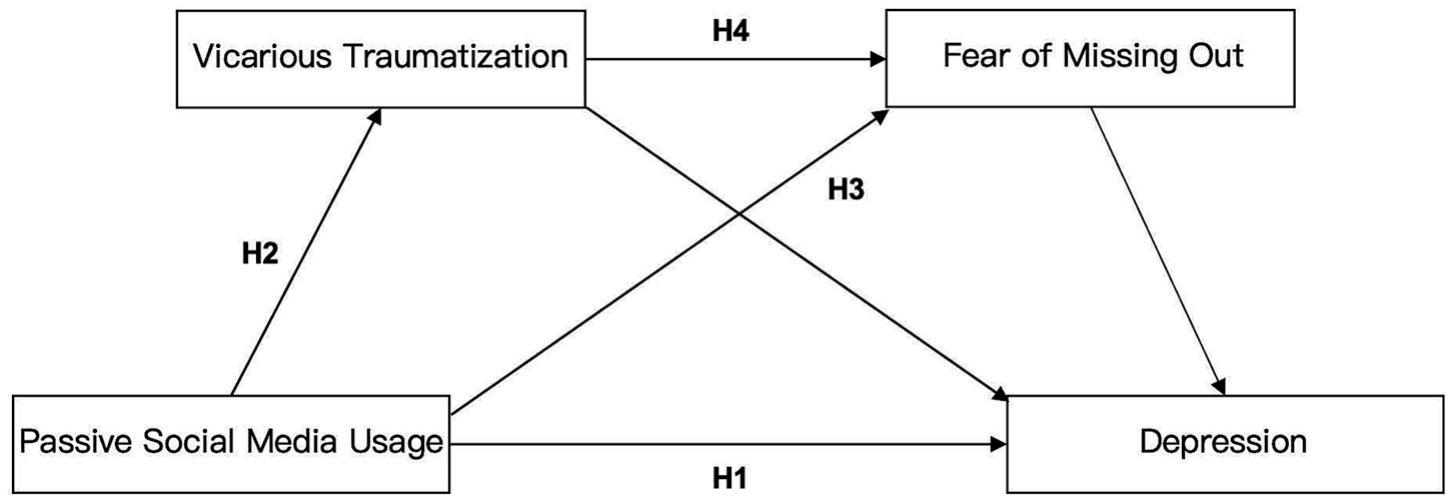
Hypothetical model.

Vicarious traumatization also influences FoMO. Research has shown that exposure to a series of adverse events heightens stress levels and negatively impacts individuals’ worldviews and values (Xu and Yang, 2009). When stressful life events become more severe, they hinder the fulfillment of fundamental psychological needs, such as competence, relatedness, and autonomy (Xia and Ye, 2014). When these psychological needs remain unmet for an extended period, individuals may seek distraction or relief by shifting their focus to a new environment. The frustration of unresolved needs can lead to heightened anxiety (Vansteenkiste and Ryan, 2013). Studies also suggest potential linkages between trauma-related psychological states and fear of missing out (FoMO), though direct evidence for vicarious traumatization (VT) remains limited. Valkenburg et al. (2022) establish that passive social media use amplifies upward social comparison, a core FoMO mechanism, particularly among individuals with pre-existing vulnerabilities. Complementarily, Zhang (2024) demonstrates that trauma symptoms (including but not limited to post-traumatic stress disorder) significantly predict FoMO, which in turn mediates social media addiction. While not explicitly measuring VT, Qutishat and Sabei (2024) reveals that nurses experiencing second victim syndrome (a construct overlapping with VT in symptomology) report heightened FoMO, with junior clinicians showing the strongest effects. Collectively, these findings imply that trauma-associated distress may sensitize individuals to FoMO triggers. During the COVID-19 pandemic, a worldwide public health crisis, college students are indirectly exposed to traumatic events by browsing pandemic-related information or reading distressing discussions on social media, leading them to experience negative emotional reactions as if they were personally affected. Individuals may feel a lack of self-efficacy, believing they are unable to control their lives or meet their psychological needs. As a result, they shift their focus to other goals, such as staying up-to-date with the pandemic, which fosters FoMO. This, in turn, may worsen their depression. Therefore, the following hypothesis could be posited by this study (see Figure 1).

*H4*: Vicarious traumatization and FoMO serve as sequential mediators in the relationship between the frequency of passive social media use and depression levels among college students.

## Materials and methods

This study employed a cross-sectional design to explore the relationship between passive social media usage and depression among college students during the COVID-19 pandemic. The design allowed for the examination of correlational associations between various psychosocial factors, such as vicarious traumatization and FoMO, and depressive symptoms without establishing causality.

A combination of questionnaire surveys and statistical analysis techniques were employed in this study to systematically collect and analyze data, ensuring a comprehensive evaluation of the research variables and their relationships. The questionnaire method enables the efficient collection of large, standardized datasets, ensuring objectivity in measurement. This resource-efficient approach saves time and promotes anonymity, which enhances the likelihood of obtaining truthful responses.

As for data analysis, this study utilized SPSS 23.0 to perform a series of statistical tests, including Analysis of Variance (ANOVA), multiple linear regression, and the Bootstrap method. These techniques allowed for a robust examination of the relationships between variables, providing both comprehensive and reliable results through parametric and non-parametric approaches. The use of the Bootstrap method further enhanced the precision of our estimates by generating confidence intervals based on resampling, ensuring the validity of our findings.

### Questionnaire design

In this study, four questionnaire scales were employed to investigate the influence of college students’ passive social media usage on their depression levels during public health emergencies. These scales include the passive social media usage scale, the vicarious traumatization scale, the fear of missing out (FoMO) scale, and the depression scale. These scales were carefully selected to comprehensively capture the key variables, enabling a deep understanding of how passive social media use and related psychological factors impact the students’ mental health during public health emergencies.

The passive social media usage scale was developed by adopting the well-established and widely used scale created by Liu et al. (2017a). Additionally, elements from the questionnaire designed by Krasnova et al. (2013), which measures the intensity of passive social media browsing behavior on Facebook, were incorporated to refine the assessment tool further. The scale was appropriately modified to adjust certain question items to align with the context of the COVID-19 pandemic. The questionnaire was reviewed by three experts in information science or psychology, along with 15 college students with social media experience. Based on their feedback, the question items were revised accordingly. Four question items were formed, including “I browse the relevant information of the COVID-19 pandemic advances,” “I read the relevant status of the COVID-19 pandemic updated by friends,” “I view the photos and videos of the pandemic situation uploaded and forwarded by friends” and “I browse group members’ ongoing conversations about the pandemic.” Each item was scored using a five-point Likert scale. In order to ensure that active social media use did not influence passive use responses, we instructed participants to answer the four relevant questions based on moments when they had no information to share or disclose while using social media. They were also asked to reflect on their passive social media usage over the past week. Higher scores on the scale indicated more frequent passive use.

The vicarious traumatization scale was developed by adopting the Chinese version of the Impact of Event Scale-Revised (IES-R) from Huang et al. (2006), along with referencing the Vicarious Traumatization Questionnaire designed by Han (2009). Classical items were modified to account for the specific context of the COVID-19 pandemic. There are 18 questions in total, such as “It is difficult for me to sleep peacefully until dawn,” “Irrelevant things remind me of the COVID-19 pandemic,” and “I feel that I am easily irritated.” A five-point Likert scale was utilized, with higher scores reflecting greater levels of vicarious traumatization.

The Fear of Missing Out (FoMO) scale was designed based on the Fear of Missing Out scale developed by Song et al. (2017), which encompasses four dimensions: psychological motivation, cognitive motivation, behavioral performance, and emotional dependence. It consists of 12 questions, such as “I am anxious when I do not access social media for a while.” A 5-point Likert scale ranging from 1 (strongly disagree) to 5 (strongly agree) was used for scoring. Higher scores indicate greater levels of fear of missing out.

For the development of the depression scale, the Center for Epidemiological Studies Depression Scale (CES-D), created by the National Institute of Mental Health (NMH) (Zhang et al., 2010), was adopted. The original scale consists of 20 items, including 4 reverse-scored items. In this study, only 16 non-reverse-scoring items were selected. Participants were asked to evaluate their feelings based on the past week, with higher scores reflecting greater levels of depression.

### Reliability and validity test

Since substantial modifications were made to the vicarious traumatization scale, particularly following expert recommendations and considering the COVID-19 pandemic context, confirmatory factor analysis (CFA) was conducted to assess the scale. The fit indicators from the analysis were found to be acceptable, indicating that the revised scale demonstrated good statistical validity: ᵪ^2^/df = 2.765, RMSEA = 0.076, IFI = 0.906, CFI = 0.905.

A reliability test is generally performed using Cronbach’s alpha reliability coefficient, which measures the internal consistency of a scale. A higher alpha coefficient indicates better consistency across items. In this study, the reliability analysis demonstrated that the Cronbach’s alpha coefficients for the passive social media usage scale, FoMO scale, vicarious traumatization scale, and depression scale were 0.864, 0.858, 0.914, and 0.945, respectively. These results indicate that the scales exhibit strong reliability and high internal consistency, confirming the robustness of the questionnaires used in this research.

### Ethical considerations

All procedures involving human participants in this study were conducted in accordance with the ethical standards of the Ethics Committee of the School of Management at Tianjin Normal University. Informed consent was obtained from all adult participants prior to their inclusion in the study. Each participant received a compensation of CNY 50 for their participation.

### Data collection

The data for this study were collected through the online questionnaire platform.^1^ College students with social media experience were invited to participate in the survey, which was conducted from March 14 to March 19, 2020, during the peak period of the COVID-19 pandemic. Participants were recruited via campus BBS, and voluntary participation was used to ensure a representative sample. Eligibility criteria required that participants be actively enrolled in a university and regular social media users. Participants were excluded if they had any clinician-diagnosed mental disorders, specifically pre-existing conditions diagnosed before the pandemic, all included participants confirmed no such history. Informed consent was obtained from all participants before they completed the questionnaire. This study employed *a priori* sample size calculations using the statistical software G*Power (version 3.1.9.4) (Faul et al., 2007) for sampling decisions before conducting the online questionnaire to ensure a sufficiently powered study and uninflated effect sizes. Calculation of the sample size for an ANOVA with the given effect size (0.05) and a power of 0.80 resulted in *N* = 269. The actual number of recruits in our study is 322.

A total of 322 questionnaires were received, with 305 deemed valid after excluding 17 responses with missing data. Of the 305 valid respondents, 240 were aged between 18 and 23, while 65 were aged 24 and above. The final sample consisted of 260 undergraduate students and 45 graduate students.

## Data analysis

This study used the five-point Likert scale to measure the key variables, with response options ranging from 1 (strongly disagree) to 5 (strongly agree). The midpoint value 3 represents a neutral or balanced position, indicating that respondents neither agree nor disagree with the statement. Using the midpoint as a reference point allows a clear interpretation of whether responses tend to skew toward agreement (scores above 3) or disagreement (scores below 3). This approach is consistent with survey design practices outlined by Chyung et al. (2017) and Nadler et al. (2015), which highlight the significance of midpoints in Likert scales for understanding participant responses. By comparing mean scores to the midpoint, we can better gauge overall tendencies in passive social media usage and fear of missing out (FoMO), providing context for how these behaviors are distributed among the participants. However, it is important to note that this comparison does not imply a strict cutoff but rather serves as an interpretive tool for understanding general trends in the data.

### Common method bias (CMB) test

To assess common method bias, we employed SPSS 23.0 to perform Harman’s single-factor test. This test involves conducting an exploratory factor analysis (EFA) without rotation to determine whether a single factor accounts for the majority of variance in the data. If a significant portion of the variance is explained by one factor, it may indicate the presence of common method bias, which could affect the validity of the results. This approach helps ensure data collection method does not unduly influence the observed relationships between variables.

### One-way ANOVA analysis

In this study, passive social media usage, vicarious traumatization, FoMO, and depression were collectively categorized as psychosocial factors, as each variable reflects aspects of individuals’ psychological well-being and social interactions. By combining these concepts, the study aimed to explore how different age groups experience and respond to these psychosocial factors, especially in public health emergencies. A one-way ANOVA was conducted to examine potential differences in these psychosocial factors across various age groups. The purpose of this analysis was to identify whether there are significant variations in the levels of passive social media usage, vicarious traumatization, FoMO, and depression among individuals of different age ranges. Significant findings would indicate that specific interventions could be designed to support vulnerable age groups in mitigating the negative effects of passive social media usage, vicarious traumatization, FoMO, and depression. On the other hand, non-significant results would suggest that these psychological factors affect individuals similarly across different age groups.

### Descriptive statistics and correlation analysis

In this study, descriptive statistical analyses were conducted on four Psychosocial Factors: Passive Social Media Usage, Vicarious Traumatization, FoMO and Depression. These analyses aimed to provide an overview of the distribution of each variable, including measures such as mean, standard deviation, and range, to capture the general trends and variations within the sample population.

Following the descriptive analysis, correlation analyses were performed to explore the relationships between these psychosocial factors. Pearson’s correlation coefficient was used to examine the strength and direction of associations between passive social media usage, vicarious traumatization, FoMO, and depression. The correlation analysis also helps provide insights into how these factors might influence one another, potentially contributing to the development of psychological interventions tailored to individuals’ needs.

### Chain mediating model test

Regression analysis was conducted to further investigate the relationship between passive social media usage and depression levels among college students. While the earlier correlation analysis indicated no significant direct relationship between these two variables, regression analysis was utilized to explore whether passive social media usage could act as a predictor of depression when controlling for additional factors, such as fear of missing out and vicarious traumatization. This approach allowed for a better understanding of how different psychosocial factors might interact to influence mental health outcomes.

A chain mediating model test was conducted to delve deeper into the complex relationships between passive social media usage and depression levels, with a specific focus on understanding the indirect pathways through two key psychosocial factors: vicarious traumatization and fear of missing out. This analysis aimed to uncover not only the direct effects of passive social media usage on depression but also the mechanisms by which these relationships unfold, particularly through the mediating influence of vicarious traumatization and FoMO. This method allows for a more comprehensive understanding of how different psychological factors interact within the context of passive social media usage, shedding light on the indirect pathways that can lead to mental health challenges.

## Results

### Common method bias (CMB) test

The results revealed nine common factors with eigenvalues greater than 1, with the first factor accounting for 29% of the variance, which is below the 40% threshold. According to the established standard (Zhou and Long, 2004), this indicates that no significant common method bias was present in this study’s sample data.

### Descriptive statistics and correlation results

The descriptive statistical results, presented in Table 1, indicate that each variable was measured using a five-point Likert scale, with a midpoint value of 3 (Jamieson, 2004; Norman, 2010). The findings show that college students scored above the midpoint for both passive social media usage frequency and FoMO, with mean values of 3.57 and 3.38, respectively. These elevated scores suggest that college students engage more frequently in passive social media usage [t(304) = 11.566, *p* < 0.001] and experience relatively high levels of FoMO [t(304) = 9.985, *p* < 0.001] compared to the scale’s medium value. However, the scores for vicarious traumatization and depression were lower, averaging 1.98 [t(304) = −27.359, *p* < 0.001] and 1.59 [t(304) = −45.342, *p* < 0.001], respectively. Given the relatively high score for passive social media usage, and considering that passive browsing is the primary behavior associated with this usage (Yue et al., 2022), it can be inferred that passive social media browsing has become a prevalent approach for college students to access information during the COVID-19 pandemic. This suggests that passive engagement with social media platforms has increasingly served as a key information source for students within this period. Moreover, college students exhibited notable symptoms of fear of missing out (FoMO) on pandemic-related information. The correlation analysis (see Table 1) demonstrated that passive social media usage frequency was significantly and positively correlated with both FoMO and vicarious traumatization but showed no significant correlation with depression levels. This finding aligns with Li′s research, which similarly found no significant relationship between passive social media usage and depression levels (Li, 2018). Other results presented in Table 1 indicate that fear of missing out (FoMO) is significantly and positively correlated with both vicarious traumatization and depression levels. Furthermore, vicarious traumatization also shows a significant positive correlation with depression levels. These relationships suggest that higher FoMO is associated with increased psychological distress, as reflected in both vicarious traumatization and depression.

**Table 1:** Descriptive statistics results and correlations between four variables.

Variables	M	SD	1	2	3	4
1 Passive social media usage	3.57	0.87	1			
2 Vicarious traumatization	1.98	0.64	0.127^*^	1		
3 Fear of missing out	3.38	0.66	0.431^***^	0.345^***^	1	
4 Depression	1.59	0.54	−0.053	0.665^***^	0.250^***^	1

*p < 0.05, **p < 0.01, ***p < 0.001.

The correlation analysis results indicated that among college students, passive social media usage frequency was significantly and positively correlated with vicarious traumatization. Additionally, significant positive correlations were found between any two variables of vicarious traumatization, fear of missing out (FoMO), and depression levels. These findings suggest interconnected relationships between these psychosocial factors. Given that the conditions for a mediation effect are met, the next step is to conduct a mediation analysis to determine whether vicarious traumatization or FoMO mediates the relationship between passive social media usage and depression, offering deeper insights into the underlying mechanisms of these associations.

### Chain mediating model results

The test results of the relationship between passive social media usage and depression level are shown in Table 2. There was no significant effect of passive social media usage frequency on depression among college students (*β* = −0.049, *p* > 0.05). Therefore, Hypothesis 1 is not supported.

**Table 2:** Linear regression results of passive social media usage and depression level.

Regression equation		Overall fit index			Significance of regression coefficients	
Dependent variables	Independent variables	*r*	*r^2^*	*F*	*β*	*t*
Depression	Passive social media usage	0.105	0.011	0.501	−0.049	−0.844
	Age				−0.056	−0.846
	Gender				−0.052	−0.879
	Educational background				0.092	1.428

*p < 0.05, **p < 0.01.

The absence of a direct effect between passive social media usage and depression levels could be attributed to several factors. Firstly, the quality of human-information interaction, rather than the frequency of usage, may play a more critical role in shaping depression. Engaging with meaningful, supportive, or informative content could mitigate negative emotional outcomes, whereas passive consumption of irrelevant or superficial content may not have a significant impact on depression. Secondly, government-led epidemic prevention and control efforts during the pandemic may have bolstered individuals’ sense of security and confidence, thereby alleviating overall depression levels. These interventions provided a stabilizing effect, reducing the psychological distress that might otherwise be associated with passive social media use. Lastly, drawing from the Stimulus-Organism-Response (SOR) model (Mehrabian, 1974), the informational stimuli encountered during passive social media browsing may evoke affective responses indirectly by altering users’ internal states or organismic conditions. In this framework, the cues or content encountered do not immediately cause depression but may influence the user’s emotional and cognitive processes, eventually shaping their psychological well-being. Therefore, the nonexistence of a direct relationship could be explained by the complex mediation of internal emotional and cognitive mechanisms that process these stimuli (Elhai et al., 2017; Valkenburg and Peter, 2013). Following the mediation effect test procedure proposed by Wen and Ye (2014), the relationship was treated as a masking effect, and the Bootstrap method was employed to further examine this effect. The analysis was conducted using the PROCESS macro developed by Hayes (2017). Specifically, the Bootstrap method was applied with 5,000 resampling iterations and a 95% confidence interval to test the mediating effect (Hayes et al., 2014; Zhao et al., 2010). Age, gender, and education were included as control variables to ensure the robustness of the results.

**Figure 2:**
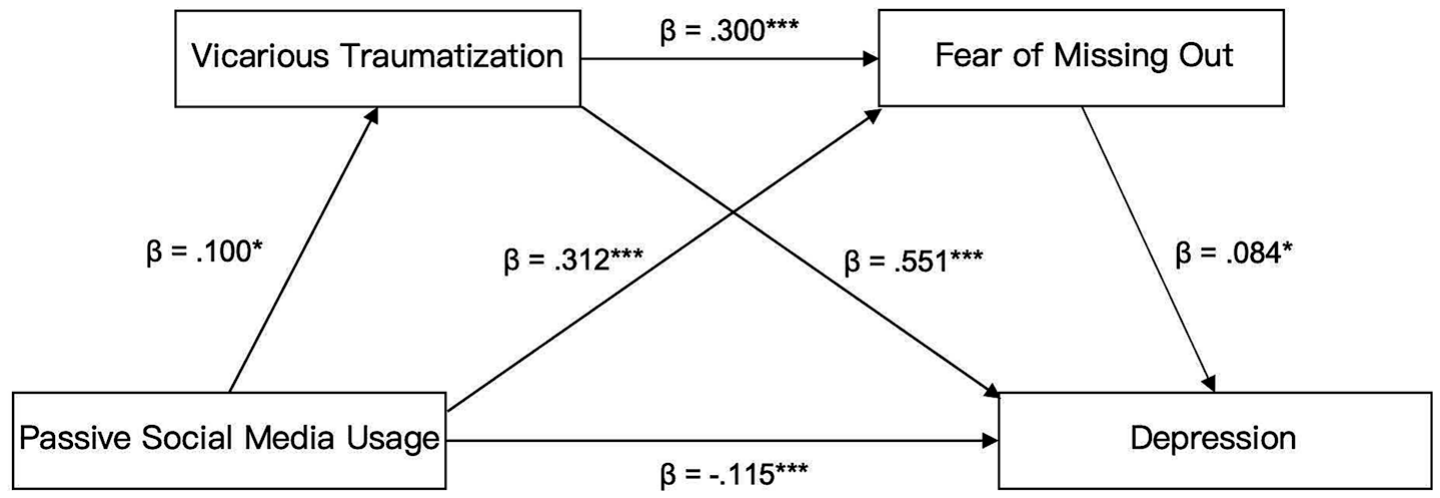
Chain mediating model.

The chain mediating effect results (Tables 3, 4) showed that the mediating effects of vicarious traumatization and fear of missing out were mainly composed of three indirect effects. (1) indirect effect 1 (mediating effect value = 0.0531): passive social media usage→vicarious traumatization→depression, Bootstrap 95% confidence interval is [0.0077, 0.0984], which does not include 0. Such result means that vicarious traumatization has a significant mediation effect on the relationship between social media usage frequency and depression level. Hypothesis 2 is thus supported. (2) indirect effect 2 (mediating effect value = 0.0258): passive social media usage frequency→fear of missing out→depression, Bootstrap 95% confidence interval is [0.0029, 0.0531] that excludes 0. Fear of missing out (FoMO) exerts a significant mediating effect on the relationship between passive social media usage frequency and depression levels among college students. Therefore, Hypothesis 3 is supported. (3) Indirect effect 3 (mediating effect value = 0.0025): passive social media usage frequency→vicarious traumatization→fear of missing out→depression, Bootstrap 95% confidence interval is [0.00001486, 0.0069], excluding 0. This provides strong evidence of a significant chain mediating effect between passive social media usage frequency and depression levels, mediated through both vicarious traumatization and fear of missing out (FoMO), thereby supporting Hypothesis 4. The indirect effects 1, 2, and 3 accounted for 27, 13.12, and 1.27% of the total effect, respectively; the total mediating effect value is 0.0813, accounting for 41.33% of the total effect (0.1967) (see Figure 2).

**Table 3:** Regression results for chain mediating model test.

Regression equation		Overall fit index			Significance of regression coefficients	
Dependent variables	Independent variables	*r*	*r^2^*	*F*	*β*	*t*
Vicarious traumatization	Passive social media usage frequency	0.175	0.031	1.897	0.100	2.29^*^
	Age				0.03	0.413
	Gender				−0.009	−0.094
	Specialty				−0.015	−0.285
	Highest education				0.148	1.412
Fear of missing out	Passive social media usage frequency	0.530	0.281	19.429	0.312	7.962^***^
	Vicarious traumatization				0.300	5.854^***^
	Age				−0.016	−0.247
	Gender				0.073	0.857
	Specialty				−0.047	−0.989
	Highest education				0.032	0.346
Depression	Passive social media usage frequency	0.685	0.47	66.402	−0.115	−3.868^***^
	Vicarious traumatization				0.551	14.566^***^
	Fear of missing out				0.084	2.077^*^
	Age				−0.068	−1.503
	Gender				−0.076	−1.272
	Specialty				−0.027	−0.816
	Highest education				0.033	0.502

^*^P < 0.05, ^**^P < 0.01, ^***^P < 0.001.

**Table 4:** Summary for chain mediating effect.

Effect type	Indirect effect size	Boot standard deviation	BootCI lower limit	BootCI upper limit	Relative mediation effect
Total indirect effect	0.0813	0.0283	0.0254	0.1363	41.33%
Indirect effect 1	0.0531	0.0233	0.0077	0.0984	27.00%
Indirect effect 2	0.0258	0.0126	0.0029	0.0531	13.12%
Indirect effect 3	0.0025	0.0018	0.0000	0.0069	1.27%

## Discussion and implications

### General discussion

This study examined the impact of passive social media usage frequency on depression among college students during the COVID-19 pandemic while also exploring the chain mediating effects of vicarious traumatization and FoMO in this relationship. The findings revealed that passive social media usage frequency did not directly predict depression. However, it significantly and positively influenced depression through the individual mediating effects of vicarious traumatization and FoMO, as well as through their combined chain mediating effects.

Results indicated that the passive social media usage frequency of college students does not have a significant impact on their depression levels (*β* = −0.049, *p* > 0.05), thus Hypothesis 1 was not supported. During the COVID-19 epidemic, social media is flooded with pandemic-related information, allowing college students to easily browse and access this content through passive social media use. However, this passive information consumption does not directly increase depression levels unless it triggers FoMO via vicarious traumatization. This finding is consistent with previous studies, which have similarly reported that the frequency of passive social media usage does not have a direct predictive effect on depression levels (Chhatwani et al., 2023). Findings from Lewin et al.’s (2023) research showed that passive social media usage was even found to be associated with lower depression levels among older adults. Meier and Krause (2023) pointed out that the effects of passive social media usage on well-being were highly variable, significantly challenging the passive use hypothesis, which posits that passive engagement is inherently harmful to well-being (“passive” = “bad”). Therefore, the influence of passive social media usage is debatable.

Two possible explanations for the lack of a direct relationship between passive social media usage frequency and depression levels were proposed by this study. Firstly, depression is likely influenced more by the quality of user-media interactions and the emotional experiences these interactions evoke rather than the frequency of usage. Davila et al. (2012) argued that time spent on social media does not necessarily result in depression. Users often engage in both passive and active social media behaviors within a given period, and the positive effects of active use may weaken or offset the negative impact of passive use (Zhang et al., 2020). Secondly, implementing various protective measures, such as government-mandated mask-wearing and quarantine policies, and consequencing the low number of infections in China from April 2020 to November 2022, bolstered public confidence in overcoming the pandemic. These efforts helped alleviate the negative emotions typically associated with passive social media use, such as the anxiety induced by browsing pandemic-related information. According to Guo (2020), over 80% of the Chinese public maintained a positive attitude toward pandemic prevention, control, and protective measures implemented by both the Chinese government and individuals. They believe that overcoming the challenges posed by the pandemic is achievable. As a highly educated group, college students possess a rational and well-rounded understanding of the COVID-19 pandemic, allowing them to remain composed and level-headed in the face of a public health emergency. Consequently, they tend to exhibit lower levels of anxiety when exposed to negative information from social media regarding the pandemic.

This study suggested that vicarious traumatization mediates the relationship between passive social media usage frequency and depression levels among college students, thereby supporting Hypothesis 2. Vicarious traumatization is a proximal factor of environment and behavior affecting individual mental health. Neuropsychological studies have provided evidence linking brain functional connectome mechanisms to COVID-19 vicarious traumatization, offering valuable insights into the prediction of general distress (Suo et al., 2022). During the COVID-19 pandemic, many Chinese people were quarantined at home. Social network sites emerged as a key source of information for them to stay informed about the pandemic. Social media browsing has become an integral part of their daily lives. Constant exposure to a flood of negative content related to COVID-19, including news about illness and death, along with the emotional distress conveyed through text, images, videos, and other multimedia formats, led users to empathize deeply with the suffering of others. This empathetic engagement adversely affected their psychological and emotional well-being, ultimately resulting in vicarious traumatization. Researchers have demonstrated that vicarious traumatization can lead to persistent negative emotional states (Wen and Ye, 2014). Over time, these prolonged emotional disturbances may ultimately trigger the onset of depression.

This study supported Hypothesis 3, showing that the relationship between passive social media usage frequency and depression levels is significantly mediated by FoMO. This finding aligns with the bibliometric results of Çelik et al. (2023), which indicate that FoMO is strongly associated with negative affectivity and problematic social media use. Passive social media usage is a vital source of FoMO generation. As pointed out by Riordan et al. (2022), social network site (SNS) use is highly associated with FoMO and the influence of the former is moderated by the latter. The World Health Organization (WHO) highlighted that the outbreak of COVID-19 was accompanied by an “infodemic,” characterized by an overwhelming surge of mixed-quality information. This flood of content made it challenging for individuals to discern between real and false information, identify trustworthy sources, and obtain reliable guidance during the pandemic. In other words, the delayed release of authoritative information during the COVID-19 pandemic led to social media being flooded with rumors, misinformation, and disinformation (Barua et al., 2020). In the face of the infodemic, users are forced to spend considerable time browsing, filtering, and identifying accurate and relevant pandemic information. This passive social media consumption can be mentally exhausting. Additionally, FoMO on valuable information intensifies this behavior, often resulting in negative emotional outcomes such as stress and depression.

The current research indicates that vicarious traumatization and fear of missing out serve as significant chain mediators in the relationship between passive social media usage frequency and depression. This finding provides strong support for Hypothesis 4. As mentioned above, passive social media use has a significant influence on vicarious traumatization. Trippany et al. (2004) argued that vicarious traumatization can lead to various psychological issues, including anxiety, with fear of missing out being a specific manifestation of such anxiety. Additionally, vicarious traumatic events can lead to a diminished sense of self-efficacy. When an individual’s sense of self-efficacy is low, they may struggle to regulate their emotions and cognition effectively. This decline in self-efficacy will further impair an individual’s ability to self-regulate. Chai et al. (2018) suggested that the fear of missing out is a direct manifestation of impaired self-regulation. In other words, vicarious traumatization undermines self-efficacy, which, in turn, contributes to the development of FoMO. Moreover, FoMO serves as a source of negative emotions, such as depression. In this study, college students frequently browsed social media to stay informed about the COVID-19 pandemic, often encountering reports of severe traumatic events. This stressful, passive information consumption led to vicarious traumatization, which in turn heightened anxiety levels, particularly FoMO on important pandemic updates. Prolonged FoMO increased their susceptibility to depression over time.

This study focuses on the impact of passive social media use on depression among college students in the context of public health emergencies, addressing several critical gaps in the existing literature. First, unlike Lee and Cho (2019), who primarily emphasized the positive role of social media in supporting mental health, we examined the negative effects of passive information consumption, highlighting the dual-edged nature of social media use. This perspective contributes to a more balanced understanding of its broader implications. Second, we extend research on psychological mechanisms within the public health domain by drawing on the moderating frameworks of Chen et al. (2023). Specifically, we delve into the mediating roles of vicarious traumatization and fear of missing out in the relationship between passive social media use and depression, offering fresh theoretical insights into the mental health challenges faced during public health crises. Moreover, in contrast to Jiang et al. (2023) and other studies that focus on the general impact of health communication, we target college students, a demographic characterized by high-frequency social media use, and illuminate their heightened sensitivity to negative information and their vulnerability to mental health challenges. This focus sheds light on the unique psychological needs of this specific population. Finally, our study provides valuable guidance for public health interventions, emphasizing the importance of mitigating passive social media use and reducing exposure to negative content as strategies to improve students’ mental health. Contributions of this study have broad applicability across several areas. Firstly, the findings can be extended to other contexts of public health crises. Public health emergencies are often accompanied by an explosion of disinformation, misinformation, and alarming news stories. In such situations, passive social media usage can easily lead to FoMO and vicarious trauma, ultimately increasing depression levels. By addressing these mediators, it is possible to reduce depression symptoms during crises. Secondly, the findings are relevant to other youth populations. Young people, much like college students, may exhibit similar cognitive and online information behavior patterns during public health emergencies. The insights and recommendations from this study can be used to enhance online information services and social support for youth, helping to lower their risk of developing psychological disorders. Finally, the findings of this study offer value to information professionals and mental health practitioners. These insights can assist in designing better social media information services and guide future research efforts, aiding professionals in addressing the mental health challenges posed by passive social media use during public health emergencies.

### Implications

Beyond the immediate context of COVID-19, the core contribution of this study lies in identifying and empirically validating vicarious traumatization and FoMO as critical mediating mechanisms linking passive social media use to heightened depression during a large-scale crisis. The identified mediation pathway delineates three critical interventions for future pandemic responses. First, universities should establish digital resilience training to help students recognize vicarious trauma signals (e.g., emotional exhaustion from prolonged exposure to crisis content) and compulsive browsing behaviors. Second, social media platforms should implement emergency safety protocols, such as automated time limits for passive scrolling during infodemic peaks. Third, healthcare systems need to develop screening frameworks incorporating passive usage metrics to enable proactive psychological support for high trauma-exposure populations. This integrated approach addresses the pathway at educational, technological, and clinical levels to mitigate mental health risks during public health emergencies. The current research also offers significant insights for adjusting mobile information behaviors to help mitigate psychological trauma and promote positive mental health, particularly among college students during public health emergencies like the COVID-19 pandemic. Artificial intelligence and large language models (LLMs) can play a key role in identifying passive social media usage behavior and encouraging users to engage in more active, creative activities tailored to their profiles. Students should practice positive self-talk when accessing pandemic-related information to avoid becoming overwhelmed by negative emotions. Additionally, they should shift from passive to active social media use. Parents and universities must intervene and support this transformation. Additionally, students should participate in stress-relieving activities such as mindfulness and physical exercise, which are proven to reduce negative emotions. Replacement therapy also offers significant benefits. For students particularly vulnerable to vicarious traumatization, targeted psychological interventions are essential. In summary, the current research, grounded in the context of public health emergencies and enriched by theoretical and empirical insights, deepens the understanding of the psychological mechanisms driving the effects of social media and offers a robust foundation for public health interventions and crisis management strategies.

## Limitations and future directions

This study specifically examined passive social media use, scrolling through content without active interaction, because this behavior dominates daily digital engagement, especially during health crises when people are flooded with pandemic news. While this study provides valuable insights into the relationship between passive social media use and depression, addressing the limitations would deepen our understanding and offer more targeted interventions to mitigate the negative psychological effects of social media use. There are several limitations that should be considered. Firstly, active social media use behavior was not examined. Active engagement with social media, such as participating in discussions, creating content, or interacting with others, has been shown to have positive effects on mental health and may help alleviate depression. Future research should investigate the balance between active and passive social media usage as well as quantify the tradeoff between the two behaviors to understand their combined influence on depression levels better. This would provide a more nuanced perspective on how different types of social media engagement affect psychological well-being. Another limitation lies in that the sample was restricted to Chinese college students, which may hinder the generalization of the findings to other demographics or cultural settings. Future research could enhance generalizability by incorporating a more diverse participant pool. Besides, this study has limitations related to the use of self-reported measures, which may introduce response bias and lack objective behavioral data. However, the self-reported method also has advantages, such as being easy to administer and able to collect a large amount of data during the emergency. Future research could mitigate these limitations by combining experimental methods and neuroscience approaches to explore causal relationships and gain deeper insights into the underlying mechanisms. Moreover, the study’s focus on the COVID-19 pandemic context may constrain extrapolation to public health emergencies with divergent epidemiological profiles or societal impacts. Future work could validate this model across diverse public health emergencies (e.g., localized outbreaks, natural disasters) and cultural contexts to establish boundary conditions and mechanism robustness. Furthermore, the current design inherently limits causal inference. To verify the causality of the pathways proposed here, we recommend longitudinal cohort studies tracking the dynamics of key variables and randomized controlled trials manipulating target variables to measure their subsequent effects on outcomes.

## Conclusion

Taking COVID-19 as a case study, this research investigated the impact of passive social media usage on depression among college students during public health emergencies, focusing on the mediating roles of vicarious traumatization and fear of missing out. The study identified significant age-related differences in passive social media use and depression levels, with younger students using passive social media less frequently but exhibiting higher susceptibility to depression. Notably, passive social media usage frequency did not directly predict depression, indicating a potential masking effect. However, the frequency of passive social media use positively predicted depression through the partial mediation of vicarious traumatization and fear of missing out. Additionally, vicarious traumatization and fear of missing out served as chain mediators collectively in the relationship between passive social media usage frequency and depression, highlighting the complex pathways through which passive social media usage influences mental health.

## Data Availability

Due to ethical restrictions and to protect participant privacy, the raw data supporting the conclusions of this article are not publicly available. However, anonymized data may be made available by the corresponding author upon reasonable request.

## Footnotes

^1^ www.wjx.cn
